# Comparison of orexigenic and anorexigenic neuropeptide levels in hyperemesis gravidarum patients with normal pregnant women: A prospective cohort study

**DOI:** 10.1097/MD.0000000000040069

**Published:** 2024-10-18

**Authors:** Mehmet Yilmaz, Şerif Aksin, Deniz Balsak, Yasmin Aboalhasan, İbrahim Batmaz

**Affiliations:** a Siirt University Faculty of Medicine, Obstetrics and Gynecology, Siirt, Turkey; b Siirt Training and Research Hospital, Obstetrics and Gynecology, Siirt, Turkey; c Mardin Artuklu University Faculty of Medicine, Obstetrics and Gynecology, Mardin, Turkey.

**Keywords:** CART, galanin, hyperemesis gravidarum, orexin, α-MSH

## Abstract

**Background:**

The aim of this study was to determine whether orexigenic neuropeptides, orexin and galanin, and anorexigenic neuropeptides, alpha-melanocyte-stimulating hormone (α-MSH) and cocaine- and amphetamine-regulated transcript (CART), are implicated in hyperemesis gravidarum (HG).

**Methods:**

Fifty pregnant women who had been diagnosed with HG between April 2022 and February 2023 at the Siirt University Faculty of Medicine Training and Research Hospital (tertiary center) were recruited for this study. An equal number of pregnant women without an HG diagnosis were included in the study as the control group. Participants’ age, pregnancy history, medical history, thyroid function test results, complete blood count results, and electrolyte levels were recorded, and their orexin, galanin, α-MSH, and CART serum levels were analyzed using an enzyme-linked immunosorbent assay.

**Results:**

No statistically significant differences in orexigenic neuropeptides (orexin and galanin) were observed between the HG and control groups. A statistical difference was found between an anorexigenic neuropeptide (α-MSH) and the control group (*P* = .012). Based on a receiver operating characteristic analysis, the α-MSH parameter was statistically significant for distinguishing between participants with an HG diagnosis and those without, with a sensitivity of 63.6%, specificity of 65.9%, and cutoff value of 11769.3 pg/mL (*P* = .012, area under curve: 0.655). Based on the severity classification of ketonuria (ketonuria levels of +1 or +2 were classified as mild, whereas levels of +3 or +4 were classified as moderate to severe), the anorexigenic CART neuropeptide was found to be a statistically significant diagnostic indicator of severe ketonuria (*P* = .020).

**Conclusion:**

α-MSH and CART levels were found to be related in HG patients and in HG patients with severe ketonuria.

## 1. Introduction

Hyperemesis gravidarum (HG) is a condition of unknown etiology characterized by intractable vomiting during pregnancy, leading to weight loss and volume depletion, which can result in ketonuria and/or ketonemia.^[1,2]^ HG is a common phenomenon in pregnant women, and it significantly impairs their quality of life.

The prevalence of HG varies. It is estimated to occur in approximately 0.3% to 3% of pregnancies, and this variation is due to differences in diagnostic criteria and ethnic differences in various study populations.^[3]^ In the United States, HG is the leading cause of hospitalization in the first half of pregnancy, second only to preterm labor as a cause of pregnancy hospitalizations in general.^[4]^

The latest guidelines from the American College of Obstetricians and Gynecologists regarding nausea and vomiting during pregnancy highlight the absence of a universally accepted definition of HG. Nevertheless, HG generally refers to severe nausea and vomiting during pregnancy. The most cited criteria for an HG diagnosis include persistent vomiting unrelated to other causes, ketonuria, electrolyte imbalance, acid–base disturbances, and significant weight loss, usually defined as the loss of at least 5% of body weight.^[5]^

The pathophysiology of HG remains an actively researched subject, with no single mechanism identified as the sole cause. Various theories have been proposed to elucidate the pathogenesis of this condition. Some studies propose that there is a genetic predisposition, as women with first-degree relatives who have experienced HG are at an elevated risk of developing HG.^[6]^ Another theory implicates changes in hormone levels during pregnancy, with some studies reporting a strong correlation between elevated human chorionic gonadotropin concentrations and HG.^[7,8]^

Regulated by a complex hypothalamic neuropeptide system that interacts with central and peripheral signals to modulate individual responses to food intake, appetite involves a complex neural network.^[9,10]^ These neuropeptides, which are released from both the central nervous system and peripheral organs and play an important role in regulating energy balance and appetite, include orexigenic (appetite-increasing) neuropeptides, such as orexin and galanin, and anorexigenic (appetite-reducing) neuropeptides, such as alpha-melanocyte-stimulating hormone (α-MSH) and cocaine- and amphetamine-regulated transcript (CART).^[11]^ Orexigenic neuropeptides play a critical role in maintaining energy balance and promoting food intake, allowing energy reserves to be replenished. Orexin, in particular, is a neuropeptide found in the hypothalamus that regulates alertness, appetite, and energy expenditure. Galanin has similarly been associated with increasing energy intake and regulating energy balance. On the other hand, anorexigenic neuropeptides such as α-MSH and CART suppress appetite, reducing energy intake and increasing energy expenditure.^[12–15]^

Although serious changes in energy and appetite metabolism occur during pregnancy, the effects of these neuropeptides in HG, a disease involving serious changes in energy and appetite metabolism, have never been investigated. Such effects may help us understand the mechanisms underlying the symptoms of nausea, vomiting, appetite, and energy balance in HG.

In this context, the aim of this study was to examine how specific neuropeptides, such as orexin, galanin, α-MSH, and CART, interact at the hypothalamic level and how this interaction contributes to the pathophysiology of HG. A better understanding of the roles of these neuropeptides in HG may enable the development of new therapeutic strategies for the management of this condition.

## 2. Materials and methods

### 2.1. Participants and interventions

Fifty pregnant women diagnosed with HG at a tertiary university hospital between April 2022 and February 2023 were recruited for this study. The study was approved by the Siirt University Ethics Committee in Turkey (2022/05.04) and was performed in accordance with the Declaration of Helsinki standards.

The control group comprised an equal number of healthy pregnant women with similar characteristics with respect to age, parity, body mass index, and gestational age. The members of the control group had no maternal comorbidities, had no ketonuria, and exhibited no vomiting or weight loss. Data on the following were recorded for both groups: age, pregnancy history, and medical history. Hemograms, electrolyte levels, and thyroid function test results were collated for all participants.

Whole blood samples were collected from all participants, and the levels of the orexigenic hormones, orexin and galanin, and the anorexigenic hormones, α-MSH and CART, were examined. The inclusion criteria for study participants in the HG group were as follows: a 5- to 20-week pregnancy, diagnosis of HG, vomiting more than 3 times a day, and weight loss of 5% or more during pregnancy, with detection of ketonuria. The exclusion criteria included the following conditions: food poisoning, chronic intestinal diseases (Crohn disease, colitis ulcerosa, etc), excessive alcohol consumption, stomach and intestinal infections, eating disorders (e.g., anorexia and bulimia), disorders such as celiac disease or lactose intolerance, and neurological conditions such as meningitis, brain tumors, and brain trauma, migraine, appendicitis, and chemotherapy drug use. In the HG group, subgroups were created based on scores on the 24-hour Pregnancy-Unique Quantification of Emesis and Nausea (PUQE-24) scale (Ebrahimi, N et al 2009) and the severity classification of ketonuria, resulting in 2 groups labeled mild and moderate to severe. Enzyme-linked immunosorbent assay analyses were then performed. For clinical comparison purposes, vomiting 7 or more times a day was classified as moderate to severe, whereas vomiting fewer than 6 times a day was classified as mild, following the PUQE-24 classification. For biological comparison, ketonuria levels of +1 (5–15 mg/dL) or +2 (15–30 mg/dL) were classified as mild, whereas levels of +3 (30–60 mg/dL) or +4 (>60 mg/dL) were classified as moderate to severe.

Blood samples of approximately 2 cc were collected from the antecubital vein of each participant to analyze the serum levels of orexin, galanin, α-MSH, and CART. These adult blood samples were centrifuged at 4000 g for 10 minutes to separate out the serum, which was then stored at −80°C in 1.5-mL Eppendorf tubes. Orexin, galanin, α-MSH, and CART serum levels were measured using a human orexin, galanin, α-MSH, and CART enzyme-linked immunosorbent assay kit (Bostonchem, USA, catalog no: BLS-1465Hu, BLS-1756Hu, BLS1924Hu, BLS0678Hu, with inter- and intra-assay calculation values of <10% and <8%, respectively).

### 2.2. Sample size

The power (1 − β) and margin of error (α) were determined in our study by calculating the sample size and the prevalence of HG. The power was planned to be at least 95% = DEFF*Np(1-p)]/[(d^2^/Z^2^_1-α/2_*(N-1) + p*(1-p)].

### 2.3. Statistical analysis

SPSS version 26 was used for statistical analyses. Normal distribution was assessed with tests such as the Kolmogorov–Smirnov or Shapiro–Wilk test. Descriptive statistics included mean ± standard deviation and median (min–max) for continuous data and frequency/percentage for categorical variables. Student *t* test was used to compare normally distributed continuous data, whereas the Mann–Whitney *U* test was used to compare nonparametric data between the HG and control groups. Receiver operating characteristic (ROC) and Youden J analyses were used to determine the index cutoff value. A *P* value was <.05 was considered statistically significant.

## 3. Results

**Figure 1.**
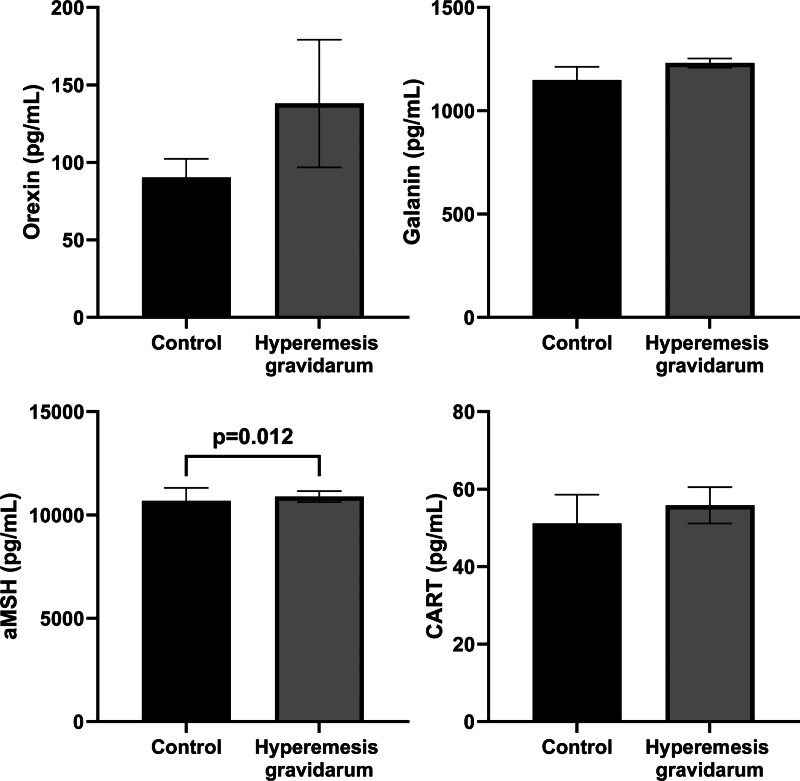
Comparing orexigenic (orexin and galanin) and anorexigenic (aMSH and CART) parameters among hyperemesis gravidarum and control groups.

**Figure 2.**
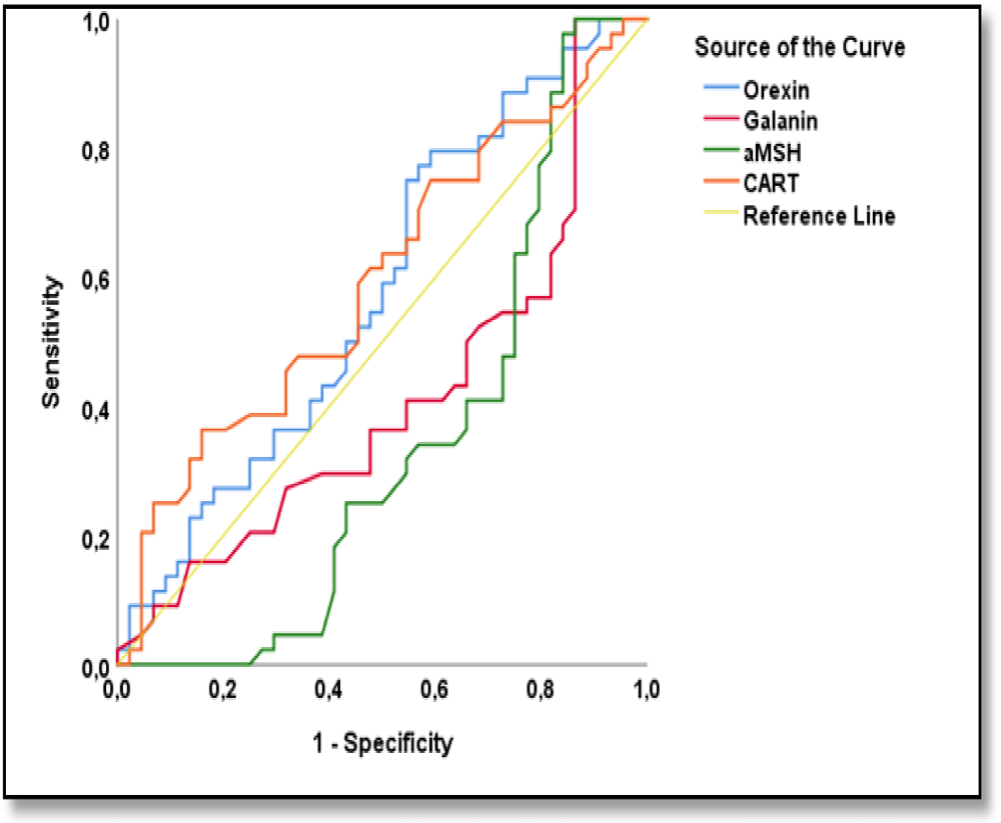
Predicting hyperemesis gravidarum vs control with orexigenic (orexin and galanin) and anorexigenic (aMSH and CART) neuropeptides; ROC analysis.

**Figure 3.**
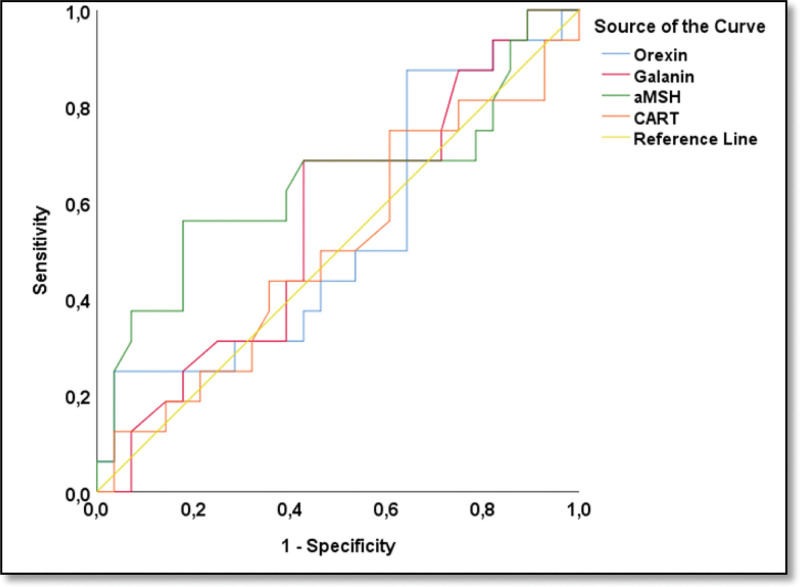
Hyperemesis classification and predicting mild and moderate&severe symptoms groups.

The clinical and biochemical parameters of the HG and control groups are compared in Table 1. The HG group and the control group were evaluated for predictive diagnostic markers among the orexigenic neuropeptides, orexin and galanin, and the anorexigenic neuropeptides, α-MSH and CART (Table 2 and Fig. 1). Based on the ROC analysis, only the α-MSH parameter was statistically significant. With the α-MSH parameter, HG cases were distinguished from members of the control group with 63.6% sensitivity, 65.9% specificity, and a cutoff value of 11769.3 pg/mL (*P* = .012, area under curve [AUC]: 0.655) (Fig. 2). In the HG group, based on the PUQE scores, the group was subdivided into 2 subgroups: mild and moderate&severe. Also, in the HG group, based on the ketonuria levels, the group was subdivided into 2 subgroups: level 1&2 and 3&4. The potential of orexigenic neuropeptides (orexin and galanin) and anorexigenic neuropeptides (α-MSH and CART) to be indicative of the difference in HG severity between these 2 subgroups was assessed using ROC analysis (Fig. 3). At this stage, there was no statistically significant predictive result for the orexin, galanin, α-MSH, and CART parameters. However, it is worth noting that, based on the Youden J analysis and ROC analysis, the highest common intersection of sensitivity and specificity was observed in the α-MSH parameter (Table 3.). This finding may contribute to the decision support phase and should be considered in the evaluation stage.

**Table 1:** Comparing parameters among hyperemesis gravidarum and control groups.

Variables	Control (n = 50)				Hyperemesis gravidarum (n = 50)				*P*-value
	Mean ± SEM	Median	Min.	Max.	Mean ± SEM	Median	Min.	Max.	
Age	26.6 ± 0.7	26	19	38	26.9 ± 0.8	26	17	41	.816
Number of vomiting/day	0.6 ± 0.2	0	0	5	6.7 ± 0.4	6	3	12	***<.0001***
BMI (kg/m^2^)	24.7 ± 0.6	23.5	19	35	24.1 ± 0.5	24	17	33	.465
Parity (n)	1.2 ± 0.2	1	0	3	1.2 ± 0.2	1	0	5	.683
Pregnancy week	10.4 ± 0.3	10	7	14	10.5 ± 0.4	10	6	15	.893
Beta-HCG (U7L)	104240.4 ± 5222.2	99,159	32,543	193,873	104497.6 ± 8251.1	91454.5	14,710	269,072	.491
TSH (mlU/mL)	1.1 ± 0.1	0.865	0.02	3.4	1.1 ± 0.2	0.79	0	5.96	.321
T3 (pg/mL)	3.1 ± 0.1	3.11	2.16	5.6	3.2 ± 0.1	3.11	1.9	5.62	.728
T4 (ng/dL)	1.1 ± 0.04	1.035	0.74	2.6	1.2 ± 0.04	1.1	0.79	2.36	***.041***
Orexin (pg/mL)	90.3 ± 12.0	64.0	13.8	428.4	138.0 ± 41.2	75.485	27.57	1848.2	.211
Galanin (pg/mL)	1148.2 ± 64.6	1302.1	24.2	1416.7	1230.8 ± 22.1	1263.0	656.3	1447.9	.171
aMSH (pg/mL)	10673.4 ± 634.7	12141.8	6.12	14428.5	10886.0 ± 267.6	11138.0	4373.9	13046.8	***.012***
CART (pg/mL)	51.1 ± 7.5	40.6	17.8	333.3	55.8 ± 4.7	44.745	19.2	171.0	.124
WBC (µL)	8.9 ± 0.3	9	5	14	9.0 ± 0.4	8	5	20	.777
Lymphocyte (%)	22.6 ± 0.6	22	14	34	22.3 ± 1.4	21	6	53	.812
Neutrophil (%)	69.7 ± 1.0	70.5	36	80	71.0 ± 1.5	72	39	91	.462
Hemoglobin (g/dL)	12.2 ± 0.2	12	10	14	12.5 ± 0.2	13	9	15	.257
Hematocrit (%)	37.8 ± 0.3	38	31	43	37.5 ± 0.5	37.5	27	44	.703
Platelet (cell/mL)	269.4 ± 7.6	273.5	182	429	279.6 ± 7.6	271.5	194	427	.346
Glucose (mg/dL)	91.8 ± 1.6	91	58	126	97.8 ± 2.8	95	68	171	.063
Urea (mg/dL)	18.0 ± 0.6	18	7	27	19.3 ± 1.5	18	7	83	.939
Creatinine (mg/dL)	0.6 ± 0.01	0.6	0.5	0.8	0.6 ± 0.01	0.605	0.45	0.9	.771
LDL (mg/dL)	96.5 ± 3.8	92	51	231	86.5 ± 3.9	77.5	34	162	.068
HDL (mg/dL)	64.7 ± 1.7	64	45	96	57.4 ± 2.0	57	34	86	***.006***
AST (U/L)	17.0 ± 0.6	16.5	12	29	20.5 ± 1.7	18	10	95	***.034***
ALT (U/L)	14.9 ± 0.8	14	8	36	16.1 ± 2.2	13	6	119	.734
Ketones in urine (%)	0 ± 0	0	0	0	1.9 ± 0.1	2	1	4	***<.0001***
CRP (mg/dL)	5.2 ± 0.5	4	1	21	5.8 ± 1.1	3	1	49	.215
Na (mmol/L)	136.0 ± 0.3	136	131	141	136.5 ± 0.4	137	128	145	.318
K (mmol/L)	3.9 ± 0.03	3.8	3.5	4.4	3.9 ± 0.04	3.8	3.3	4.8	.720
Ca (mg/dL)	9.2 ± 0.04	9.2	8.5	10	9.3 ± 0.1	9.3	7.7	10.2	.471
Klor (mmol/L)	105.2 ± 0.3	105.5	101	109	104.8 ± 0.4	105	92	111	.384

Independent *t* test and Mann–Whitney *U* test were used. *P* < .05 considered significant.

ALT = alanineaminotransferase, AST = aspartataminotransferase, BMI = body mass index, CART = cocaine- and amphetamine-regulated transcript, CRP = C-reactive protein, TSH = thyroid stimulating hormone, WBC = white blood cell, α-MSH = alpha-melanocyte-stimulating hormone.

**Table 2:** Predicting hyperemesis gravidarum with orexigenic (orexin and galanin) and anorexigenic (aMSH and CART) neuropeptides.

Variables	AUC	Std. error	*P*-value	95% CI		Sensitivity (%)	Specificity (%)	Cutoff value (pg/mL)
				Lower bound	Upper bound			
Orexin (pg/mL)	0.577	0.061	.211	0.457	0.698	59.1	50.0	61.82
Galanin (pg/mL)	0.585	0.062	.171	0.293	0.537	59.1	61.4	1283.9
aMSH (pg/mL)	0.655	0.061	***.012***	0.226	0.464	63.6	65.9	11769.3
CART (pg/mL)	0.595	0.061	.124	0.476	0.714	59.1	54.5	43.1

ROC analysis was used and *P* < .05 considered significant.

AUC = area under curve, CART = cocaine- and amphetamine-regulated transcript, CI = confidence interval, α-MSH = alpha-melanocyte-stimulating hormone.

**Table 3:** Predicting mild and moderate&severe symptoms (PUQE) in hyperemesis gravidarum with orexigenic (orexin and galanin) and anorexigenic (aMSH and CART) neuropeptides.

Variables	AUC	Std. error	*P*-value	95% CI		Sensitivity (%)	Specificity (%)	Cutoff value (pg/mL)
				Lower bound	Upper bound			
Orexin (pg/mL)	0.533	0.093	.714	0.351	0.716	50.0	46.4	70.5
Galanin (pg/mL)	0.560	0.090	.510	0.385	0.736	68.0	50.0	1208.3
α-MSH (pg/mL)	0.640	0.095	.127	0.452	0.827	68.8	57.1	10980.1
CART (pg/mL)	0.506	0.093	.951	0.324	0.688	50.0	50.0	44.8

ROC analysis was used and *P* < .05 considered significant.

AUC = area under curve, CART = cocaine- and amphetamine-regulated transcript, CI = confidence interval, α-MSH = alpha-melanocyte-stimulating hormone.

**Figure 4.**
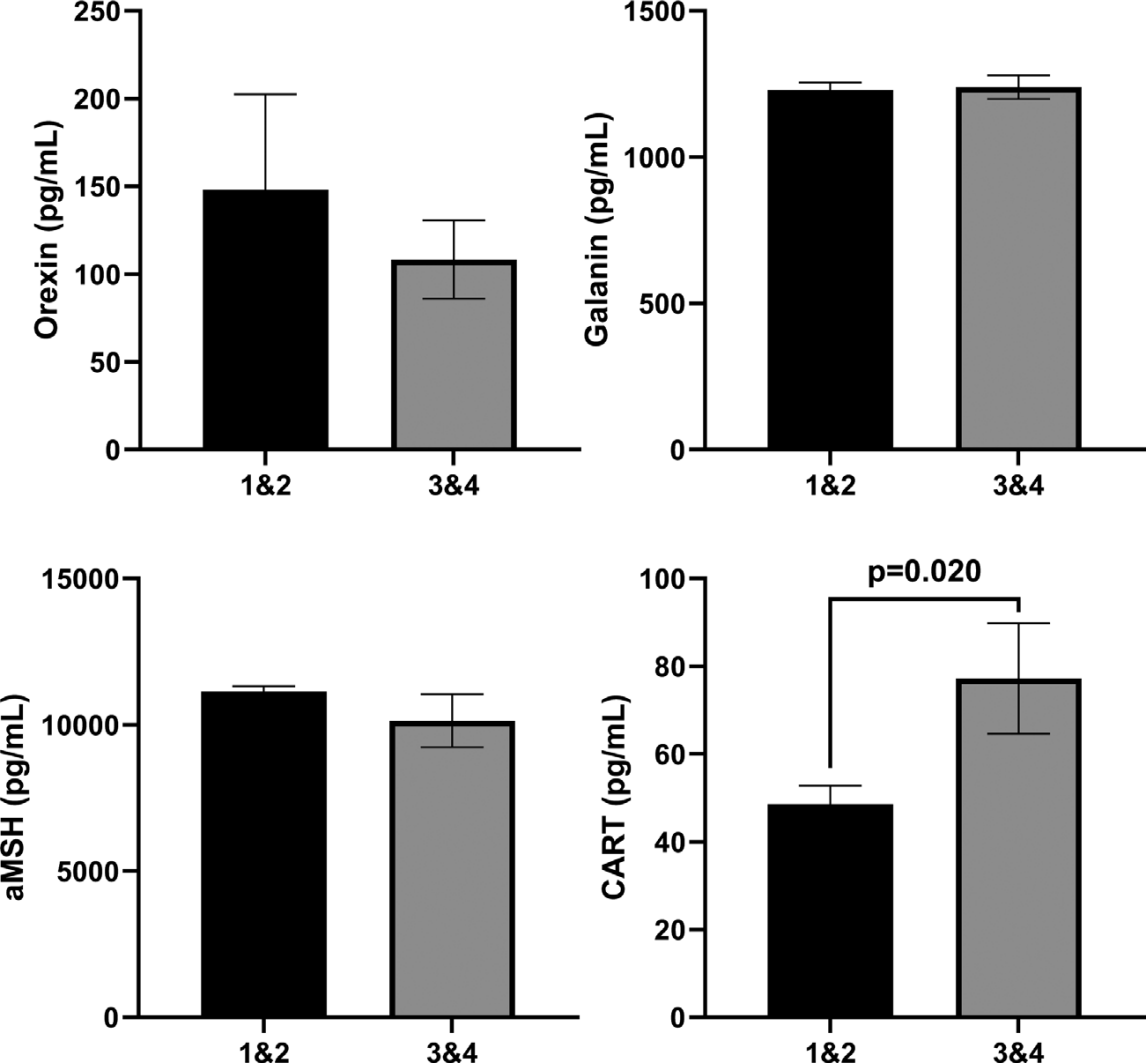
Comparison of orexigenic (orexin and galanin) and anorexigenic (aMSH and CART) neuropeptide measurements among severity classes of ketonuria in the hyperemesis gravidarum (HG) group.

**Figure 5.**
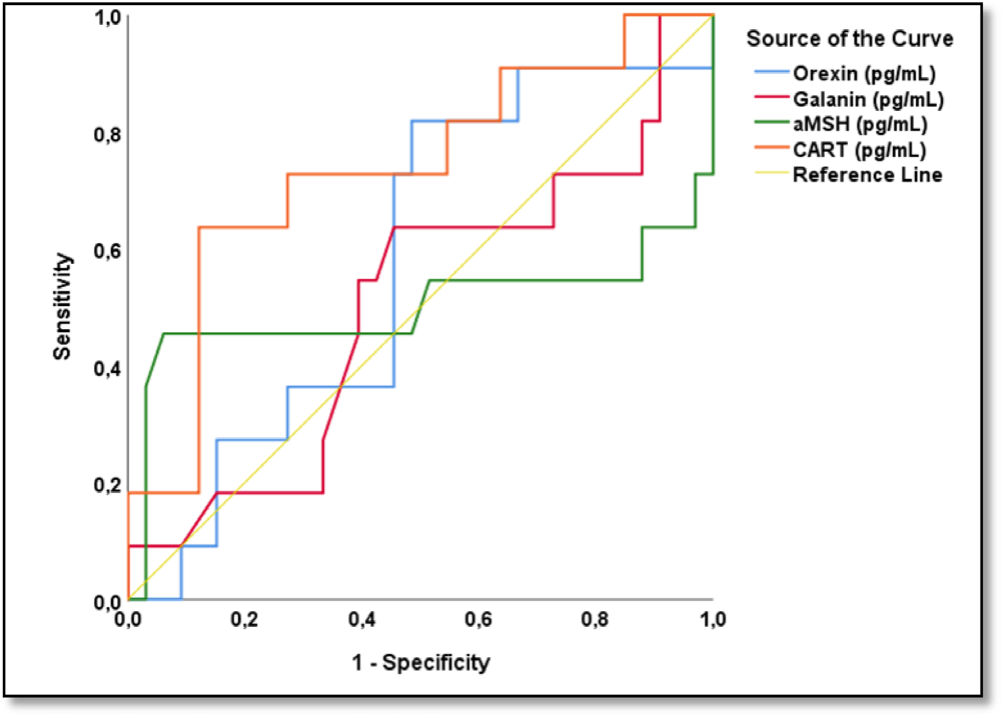
Predicting ketonuria mild and moderate&severe levels in hyperemesis gravidarum with orexigenic (orexin and galanin) and anorexigenic (aMSH and CART) neuropeptides.

In the HG group, based on the classification of ketonuria severity as level 1&2 and 3&4, the group was subdivided into 2 distinct subgroups to assess whether orexigenic neuropeptides (i.e., orexin and galanin) and anorexigenic neuropeptides (i.e., α-MSH and CART) can serve as markers for measuring the severity of HG via ROC analysis (Table 4. and Fig. 4). Based on the analysis results, there were no statistically significant predictive results for the parameters orexin, galanin, and α-MSH (*P* > .05). However, it is noteworthy that there was a statistically significant result, based on the ROC analysis and Youden J analysis, for the CART parameter, which had the highest common intersection of sensitivity and specificity (Table 5 and Fig. 5). This finding may contribute to the decision support phase and should be considered in the evaluation stage. A statistically significant difference was found in the HG group when comparing the ketonuria levels (1&2 and 3&4) to the mild and moderate&severe groups obtained from the PUQE score (*P* < .001; X2 = 15.40). The proportion of patients with a common intersection between ketonuria 3&4 level and moderate&severe group according to PUQE score remained at 57.1%, indicating that the grading of the severity of HG was successful in favor of ketonuria.

**Table 4:** Comparison orexigenic and anorexigenic parameters among ketonuria level groups in hyperemesis gravidarum.

Variables	Ketonuria groups		*P*-value
	(+1 & 2) (n = 33)	(+3 & 4)(n = 11)	
	Mean ± SD		
Orexin (pg/mL)	147.9 ± 313.7	108.3 ± 73.9	0.440
Galanin (pg/mL)	1280.0 ± 152.6	1239.1 ± 134.5	0.957
aMSH (pg/mL)	11135.2 ± 1085.6	10138.5 ± 2993.5	0.989
CART (pg/mL)	48.6 ± 24.0	77.2 ± 41.8	***0.020***

Mann–Whitney *U* test used and *P* < .05 considered significant.

CART = cocaine- and amphetamine-regulated transcript, α-MSH = alpha-melanocyte-stimulating hormone.

**Table 5:** Predicting ketonuria level 1&2 and level 3&4 in hyperemesis gravidarum with orexigenic (orexin and galanin) and anorexigenic (aMSH and CART) neuropeptides.

Variables	AUC	Std. error	*P*-value	95% CI		Sensitivity (%)	Specificity (%)	Cutoff value (pg/mL)
				Lower bound	Upper bound			
Orexin (pg/mL)	0.579	0.096	.440	0.390	0.767	72.7	45.5	70.5
Galanin (pg/mL)	0.506	0.105	.957	0.300	0.711	54.5	60.6	1276.0
aMSH (pg/mL)	0.499	0.137	.989	0.231	0.767	45.5	93.9	12556.1
CART (pg/mL)	0.736	0.092	***.020***	0.555	0.916	***63.6***	***87.9***	***68.0***

ROC analysis was used and *P* < .05 considered significant.

AUC = area under curve, CI = confidence interval.

## 4. Discussion

Understanding appetite regulation is crucial for comprehending the etiology of HG. α-MSH, a key anorexigenic peptide, influences food intake and energy expenditure by interacting with various neuropeptides. In our study, elevated α-MSH levels in the HG group impacted feeding behavior and the food-related reward system.^[16]^ Another peptide, CART, is proposed to have multiple functions, including regulating feeding, appetite suppression, addiction, stress, anxiety, innate fear, neurological diseases, neuropathic pain, depression, osteoporosis, insulin secretion, thirst, body temperature, learning, memory, reproduction, vision, and sleep regulation. The CART peptide interacts with numerous neuropeptides, such as α-MSH, and its functions are still being explored.^[17]^

In our study, only α-MSH showed statistical significance in comparing the HG and control groups, with a sensitivity of 63.6%, specificity of 65.9%, and cutoff value of 11769.3 pg/mL according to ROC analysis (*P* = .012, AUC: 0.655).

Landyman et al conducted a study on pregnant and nonpregnant rats and reported that the pregnant rats exhibited weaker responses at the hypothalamic level following α-MSH injection compared to the nonpregnant rats.^[18]^ They proposed that further investigation is needed to understand the unresponsiveness to α-MSH that underpins appetite regulation or increased appetite during pregnancy. In our study, we also observed elevated α-MSH levels in the HG group. A conceivable explanation for the increased appetite observed in normal pregnancies is that the weight loss and loss of appetite HG patients experience may be linked to increased α-MSH levels in a manner analogous to a similar connection in nonpregnant female populations. However, the reason for the elevated α-MSH levels in the HG group remains unclear. During pregnancy, a positive energy balance state develops to support the growing fetus and prepare the mother’s body for the metabolic requirements of lactation. As part of this maternal adaptation, the satiety response to α-MSH is suppressed. However, whether this suppression occurs at the receptor level or via other underlying mechanisms is uncertain.^[18]^

In the same study by Landyman et al, the melanocortin receptor mRNA levels in pregnant rats were similar to those in nonpregnant rats. This indicates that receptor downregulation may not be the mechanism responsible for the decreased response to α-MSH during pregnancy, and different mechanisms may be involved.^[19]^

It is unknown whether the elevated α-MSH levels in the HG group were due to excessive responses resulting from the physiological hypothalamic insensitivity that develops during pregnancy. Studies investigating the responses of hypothalamic nuclei in HG and non-HG groups may provide guidance.^[20]^

Li et al reported that inflammation-induced proteins, such as toll-like receptor 4 (TLR4), increase the expression of melanocortin-4 receptor and pro-opiomelanocortin (POMC), thus activating POMC neurons. They proposed that an increase in POMC leads to increased α-MSH levels, suppressing appetite.^[21,22]^ Research has linked increased TLR4 levels to the underlying mechanisms behind many pregnancy-related diseases. TLR4 has been reported to be released by various cells in the first trimester, and the induced proinflammatory environment is produced by a physiological process.^[23,24]^ It has been reported that HG patients have an elevated proinflammatory environment compared to normal pregnant women. This elevated proinflammatory environment may cause an increase in α-MSH levels due to factors such as TLR4, possibly impacting appetite and eating behavior. Further research is needed in this regard.^[25,26]^

In our study, among the ketonuria subgroups in the HG group (mild: +1, +2; moderate to severe: +3, +4), only the CART parameter showed a significant increase in the moderate to severe subgroup (*P* = .020); no significant differences were observed in other parameters. A statistically significant difference was found in the HG group when comparing the ketonuria levels (1&2 and 3&4) to the mild and moderate&severe groups obtained from the PUQE score (*P* < .001; X2 = 15.40). The proportion of patients with a common intersection between ketonuria 3&4 level and moderate&severe group according to PUQE score remained at 57.1%, indicating that the grading of the severity of HG was successful in favor of ketonuria.

Previous studies have reported that CART hormone expression increases in response to dehydration, osmotic changes, and alterations in extracellular sodium and water levels in the body to maintain a negative energy balance.^[27,28]^ Similarly, it has been reported that CART expression increases in cases of decreased vascular pressure.^[29]^ In patients with HG who experience severe water loss and moderate to severe ketonuria, it is possible that the increase in CART expression occurs in response to body water loss, sodium changes, and decreased vascular tone. Further research is needed to determine whether this increase in CART expression is a cause or a consequence.

There are only a handful of human studies linking CART levels to pregnancy. Hehir et al measured serum CART levels in both diabetic and nondiabetic human pregnancies and reported that CART plays a significant role in regulating body mass during pregnancy.^[13]^ Pazos et al demonstrated that neuropeptides derived from the placenta, such as CART and POMC, are regulated by interleukin 6 in pregnant rats, and that the effects of interleukin 6 extend to the placenta (the primary organ in the fetal–maternal interface), impacting energy and weight balance during pregnancy. Another study found that CART levels change during pregnancy and are related to appetite and weight loss symptoms, correlating with the week of pregnancy.^[30]^ Valera et al found increased CART expression on the 19th day of pregnancy in female rats, indicating that this elevated CART expression may play a role in hormonal mechanisms that stimulate maternal behavior in CART neurons and may be influenced by reproductive status.^[31]^ Therefore, the comparison between the levels of ketonuria 3&4 and moderate&severe, established according to the PUQE score, resulted in significant success in classifying the severity of the disease with the CART biomarker in patients diagnosed with HG.

Our study has limitations. First, the small sample size (100 participants) may limit the statistical power. Second, the study was conducted exclusively at a tertiary university hospital, which raises questions about generalizability to diverse populations. The strict exclusion criteria may have reduced the representativeness of the general pregnant population. Third, the focus on specific neuropeptides narrowed the research scope, excluding consideration of other potentially relevant biomarkers. Fourth, neuropeptide levels were examined at only 1 stage of pregnancy without follow-up. The study’s strengths include the pioneering examination of HG and neuropeptides, the standardized PUQE-24 for severity assessment, and consideration of ketonuria for understanding biological effects.

## 5. Conclusion

Our study revealed a potential association between α-MSH and CART with the development and severity of HG. However, we acknowledge that these findings are not sufficient to draw definitive conclusions that α-MSH and CART play a critical role in HG. Instead, we suggest that these neuropeptides may be associated with HG and that these aspects should be further investigated in future studies for a more in-depth understanding of HG. This study provides important clues about the relationship of α-MSH and CART with HG, and more comprehensive studies are required to understand the role of these neuropeptides in the pathophysiology of HG. Furthermore, considering the lack of studies directly examining the relationship between appetite regulation and HG severity, our study contributes to efforts to understand the complex pathophysiology of HG, but the effects of neuropeptides on appetite should be further investigated in the context of HG.

## Acknowledgments

We would like to thank the Siirt University Scientific Research Project Directorate for its contributions to this research (Project number: 2022-SİÜTIP-054).

## Author contributions

**Data curation:** Mehmet Yilmaz, Deniz Balsak, Yasmin Aboalhasan.

**Formal analysis:** Şerif Aksin, Deniz Balsak.

**Methodology:** Mehmet Yilmaz, Şerif Aksin.

**Software:** Mehmet Yilmaz.

**Visualization:** Mehmet Yilmaz, Deniz Balsak, Yasmin Aboalhasan, İbrahim Batmaz.

**Writing – original draft:** Mehmet Yilmaz, Şerif Aksin.
